# Lightweight cost-effective hybrid materials for energy absorption applications

**DOI:** 10.1038/s41598-022-25533-3

**Published:** 2022-12-06

**Authors:** Marwa A. Abd El-baky, Mahmoud M. Awd Allah, Madeha Kamel, Walaa Abd-Elaziem

**Affiliations:** 1 Mechanical Design and Production Engineering Department, Zagazig University, Zagazig, 44519 Egypt; 2 Mechanical Engineering Department, Suez Canal University (SCU), Ismailia, Egypt

**Keywords:** Energy science and technology, Materials science, Biomaterials, Materials for devices, Engineering, Mechanical engineering

## Abstract

The present paper experimentally explores the influence of the fiber hybridization and layering sequence on crashworthiness behavior and deformation history of polymer/metal thin-walled pipes. Jute (J)/glass (G) reinforced epoxy over wrapped aluminum (Al) pipes were prepared via hand wet wrapping then subjected to axial quasi-static compressive loads. The load versus displacement plots and crashing indicators, i.e. peak crushing load (${\mathrm{F}}_{\mathrm{ip}}$), mean crushing load (${\mathrm{F}}_{\mathrm{m}}$), total energy absorption ($\mathrm{U})$, specific energy absorption $\left(\mathrm{SEA}\right)$, and crush force efficiency $\left(\mathrm{CFE}\right)$ were determined. Experimental results revealed that the maximum $\left(\mathrm{SEA}\right)$ was recorded for Al/2J/4G/2J pipe with a value of about 42.92 kJ/g, with an enhancement of 20.56% in $\left(\mathrm{SEA}\right)$ compared with pure Al-pipes. Al/2J/4G/2J specimens display the maximum ($\mathrm{U})$, $\left(\mathrm{SEA}\right)$, and $\left(\mathrm{CFE}\right)$ and could be employed as energy absorbing members in automobiles.

## Introduction

Recently, thin-walled sections have been broadly used as crashworthy components in vehicle and rail-way industries because of their numerous profits, including high energy absorbing capability, high stiffness, high strength, high corrosion resistance, low weight, low cost, and ease of fabrication^1,2^. “Crashworthiness” can be defined as the capability of a vehicle to withstand crashes with minimal injury or damage to human bodies or goods^3,4^. Material type is an important factor that affect the crashworthy devices performance^5^. Conventionally metallic materials can be used due to the controllable plastic deformation^6^. On the contrary, polymer composites are widely utilized owing to the respectable specific stiffness and/or strength, and outstanding energy absorbing capability. Composites do not exhibit plastic deformation due to the fragility. Composite materials absorb energy by crushing and delamination^7,8^.

Hybrids have been adapted in energy absorbers as they combine plastic deformation of metallic materials and greater specific stiffness and/or strength of composites^9,10^. Many scholars examined the collapse performance of hybrid pipes. Babbage and Mallick^11^ experimentally studied the axial crushing performance of glass–epoxy overwrapped aluminum ($\mathrm{Al}$) pipes. The orientation angle of E-glass was ± 45° or ± 75° to the pipe axis. Circular and square ($\mathrm{Al}$) pipes were adapted. Some pipes were filled with epoxy foam. Results indicated that as the number of E-glass plies increase the crashworthiness parameters will be enhanced. The crashing parameters of round hybrid pipes are better than those of square ones. ± 45° orientation angle gives better crashing parameters than those of ± 75°. Kalhor and Case^12^ found that overwrapping S2-glass reinforced epoxy layers on stainless steel (St) square cylinders could alter the collapse mode from splitting with low total absorbed energy ($\mathrm{U})$ to symmetric or mixed mode with high ($\mathrm{U})$ and low oscillation in post-crash stage. The number of glass/epoxy layers in hybrid cylinders has a major effect on ($\mathrm{U})$. A new trigger mechanism was adapted which alter the failure response to a symmetric collapse mode and as a consequence it enhance crush force efficiency $\left(\mathrm{CFE}\right)$ of the proposed hybrids.

Liu et al.^13,14^ investigated the crashing behavior of carbon fiber reinforced plastic ($\mathrm{CFRP}$)/($\mathrm{Al}$) honeycomb structures under axial loading. Results indicated that peak crushing force (${\mathrm{F}}_{\mathrm{ip}}$) and ($\mathrm{U}$) of ($\mathrm{CFRP}$) filled structures are improved by 10% compared with unfilled ones. With reducing $(\mathrm{Al})$ honeycomb division length, $(\mathrm{U})$ increases gradually while $\left(\mathrm{SEA}\right)$ decreases. Crashworthiness of hybrid materials has been studied in the literature. Zhu et al.^15^ studied the crashing indicators including $(\mathrm{U})$, and the failure response of three ($\mathrm{CFRP}$)/($\mathrm{Al}$) configurations subjected to quasi-static axial loads. For comparison, empty ($\mathrm{Al}$) and ($\mathrm{CFRP}$) cylinders were tested. Experimental results indicated that H_i_ i.e., ($\mathrm{Al}$) cylinder with inner ($\mathrm{CFRP}$) cylinder achieves the best results. H_i_ was analytically studied from the viewpoints of cost and lightweight. It was reported that for the same $(\mathrm{U})$, H_i_ reduces the cost by 32.1% compared with ($\mathrm{CFRP}$) cylinder and reduces weight by 33.6% compared with ($\mathrm{Al}$) cylinder. H_i_ could be adapted for energy absorption. Sun et al.^16^ studied the quasi-static crushing performance of ($\mathrm{CFRP}$)/($\mathrm{Al}$) hybrid pipes prepared by the filament winding. It was reported that the winding angle and the specimen wall thickness have an important effect on the failure mechanism and crushing parameters. Increasing the winding angle decreases $\left(\mathrm{SEA}\right)$, $(\mathrm{U})$, and $({\mathrm{F}}_{\mathrm{ip}})$ of ($\mathrm{CFRP}$) and ($\mathrm{CFRP}$)/($\mathrm{Al}$) hybrid pipes. Increasing ($\mathrm{CFRP}$) pipe’s thickness improves $\left(\mathrm{SEA}\right)$, $(\mathrm{U})$, and $({\mathrm{F}}_{\mathrm{ip}})$ of ($\mathrm{CFRP}$) and ($\mathrm{CFRP}$)/($\mathrm{Al}$) hybrids. At 25° winding angle and 9-ply of ($\mathrm{CFRP}$), $\left(\mathrm{SEA}\right)$ of ($\mathrm{CFRP}$) and ($\mathrm{CFRP}$)/($\mathrm{Al}$) pipes were the best (48.74 and 79.05 J/g). Furthermore, $(\mathrm{U})$ of ($\mathrm{CFRP}$)/($\mathrm{Al}$) hybrid pipe exceeds the sum of its components.

According to Wang et al.^17^, the change in the deformation mode of ($\mathrm{Al}$)/($\mathrm{CFRP}$) hybrid pipes improves the internal energies $({\mathrm{U}}_{\mathrm{i}})$ of ($\mathrm{Al}$) and ($\mathrm{CFRP}$) pipes by 43.6 and 17.8% compared with pristine ($\mathrm{Al}$) and ($\mathrm{CFRP}$) pipes, respectively; and enhances the frictional dissipation energy $({\mathrm{U}}_{\mathrm{d}})$ by 45.6% compared to the sum of that of pristine ($\mathrm{Al}$) and ($\mathrm{CFRP}$) pipes, largely improving $(\mathrm{U})$ of ($\mathrm{Al}$)/($\mathrm{CFRP}$) hybrid pipes. For ($\mathrm{CFRP}$)/($\mathrm{Al}$), $({\mathrm{U}}_{\mathrm{i}})$ rises by 27.6% for ($\mathrm{Al}$) pipe but declines by 31.9% for ($\mathrm{CFRP}$) pipe compared with pristine ($\mathrm{Al}$) and net ($\mathrm{CFRP}$) pipes, respectively; whereas $({\mathrm{U}}_{\mathrm{d}})$ decreases by 47.6% compared with the sum of that of pristine ($\mathrm{Al}$) and ($\mathrm{CFRP}$) pipes, signifying the significance of hybridization on $(\mathrm{U})$. The impact of wall thickness, dimensions, and sectional shape on $(\mathrm{U})$ as well as the cost ratio of the hybrids were also considered. It was recorded that hybrid pipe with thicker ($\mathrm{CFRP}$) pipe has greater $(\mathrm{U})$; whereas hybrid with thinner ($\mathrm{Al}$) pipe exhibits better cost-effective energy absorption features. Furthermore, with constant weight, circular hybrid pipe with smaller sectional size displays the best performance. Zang et al.^18^ investigated the impact of cross-section shape on the quasi-static axial crashing of ($\mathrm{CFRP}$)/($\mathrm{Al}$) hybrid pipes. $\left(\mathrm{SEA}\right)$ and crash force efficiency $\left(\mathrm{CFE}\right)$ of the ($\mathrm{CFRP}$)/($\mathrm{Al}$) pipes with circle cross section were found to be the largest. It was figured out that the length of hybrid pipes with the circle cross section has no substantial effect on $(\mathrm{U})$, but the thickness ratio of ($\mathrm{Al}$) to ($\mathrm{CFRP}$) i.e., (t_m_/t_c_), the number of layers, the direction of fiber, and the fiber ratio in the axial/circumferential direction have noteworthy impact on the crashing behavior.

Fiber reinforced composites display good specific crashworthiness performance that, intensely depend on the constituent materials and fiber arrangement, commonly surpass those of metals^19^. On the other hand, metals offer relatively cost-efficient solutions with good understood and stable energy absorbing mechanisms^20^. Combining fiber reinforced composites and metals into hybrid systems could open new possibilities in terms of cost efficient-specific functional performance under crashing loads^20^. A properly designed metal/composite hybrid structures has been proved to be lighter and safer than traditional metals and composites with appropriate cost. This supports metal/composite hybrid structures to be adapted as an excellent substitute in crashing applications Mahdi and El Kadi^21^. In this respect, efforts have been made to choose the appropriate reinforcements in composites to absorb extra energy in progressive crushing mode. Recent researchers tried to mitigate the utilization of expensive synthetic fibers by adapting cheap, biodegradable, lightweight natural fibers Supian et al.^22^. Several studies have been carried out to explore the crashworthiness of natural fiber reinforced composites^23–26^.

Due to their exceptional multipurpose and crashworthy qualities, metal-composite hybrid constructions have become more popular in the automotive industry. Many studies on crashworthiness of metal-synthetic fiber composite hybrids were found in the literature. However, very few works have focused on revealing energy absorption mechanisms of metal-synthetic fiber composite-natural fiber composite hybrid structures; and how to control the performance to cost qualities of these structures is still an unsolved issue. This study aims to reduce the cost and increase the energy absorption of different configurations i.e., aluminum ($\mathrm{Al}$)/jute ($\mathrm{J}$)/E-glass ($\mathrm{G}$) reinforced epoxy hybrid pipes. The impacts of the reinforcement hybridization process and the layering stacking sequences have been investigated. Specimens were made-up via wet warping by hand lay-up procedure and tested under quasi-static axial loads. The crashworthiness indicators were determined, and the deformation history was examined. The cost ratio for the proposed energy dissipating elements was calculated and compared. Scanning electron microscopic (SEM) images were included to show the failure signs in the failed specimens.

## Experimental work

### Constituents

**Figure 1:**
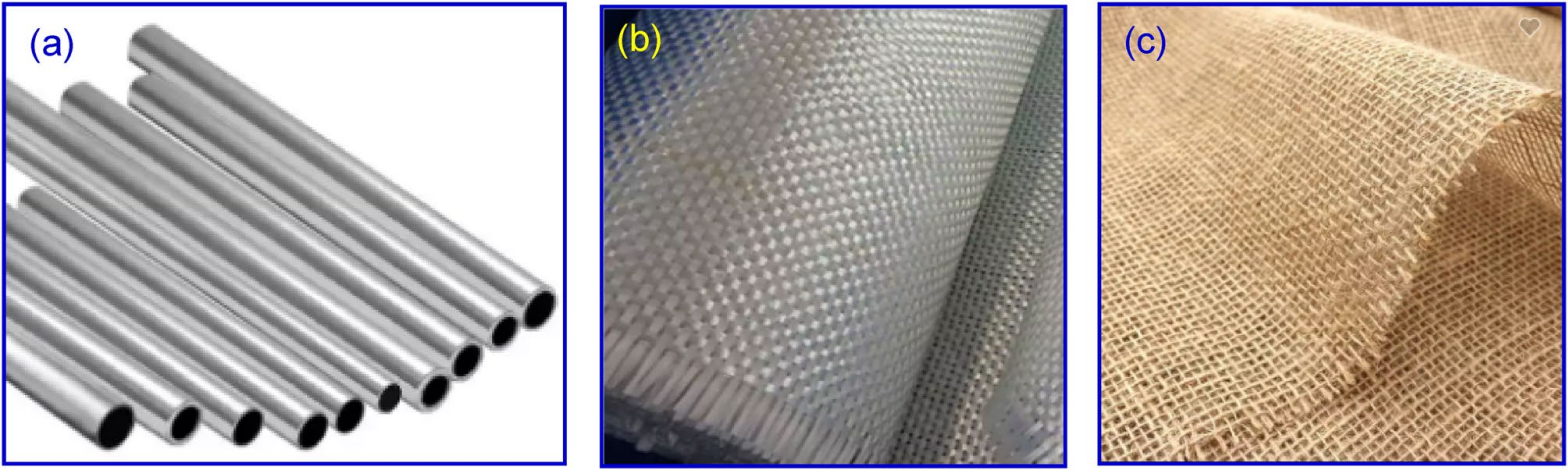
Surface images of (**a**) Al 6063 pipes, (**b**) E-glass fabric woven fabric, and (**b**) woven jute fabric.

($\mathrm{Al}6063$) aluminum alloy, supplied by Military Production Co. Ltd. (Egypt), in the form of pipes with 50 mm outer diameter and 2 mm thickness was adapted in this work. Woven E-glass and jute fabrics with 200 g/m^2^ areal densities were supplied by Hebei Yuniu Fiber Glass Manufacturing Co. Ltd., China and Zhong Xing Cotton and Jute Co. Ltd. (China), respectively. Surface images of ($\mathrm{Al}6063$) pipes, jute fabric, and E-glass woven fabric are shown in Fig. 1. Kemapoxy 150RGL delivered by Chemicals for Modern Buildings Co. Ltd. (Egypt) was nominated as a matrix. Table 1 demonstrates the mechanical characteristics of E-glass, jute, $\mathrm{Al}6063$, and Kemapoxy 150 RGL. The chemical composition of $\mathrm{Al}6063$ (weight percentage, wt%) is stated in Table 2.

**Table 1:** The properties of reinforcements and matrix given by the supplier.

Property	E-glass	Jute	Al6063	Matrix
Density, g/cm^3^	2.5	1.35	2.7	1.1
Young’s modulus, GPa	76	17	68.9	1.2
Elongation, %	1.8–3.2	1.5–1.8	18–33	2.2–2.9
Tensile strength, MPa	3400	470	186	55–58

**Table 2:** Al 6063 chemical composition (weight percentage, wt%).

Si	Fe	Cu	Mn	Mg	Zn	Ti	Al
0.44	0.18	0.01	0.04	0.48	0.01	0.01	Bal

### Specimens’ manufacturing

**Figure 2:**
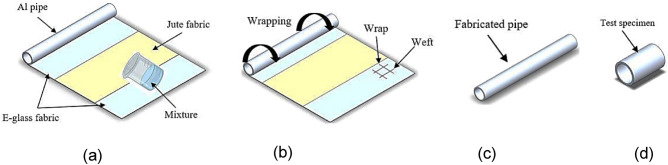
Manufacturing process sequence.

**Figure 3:**
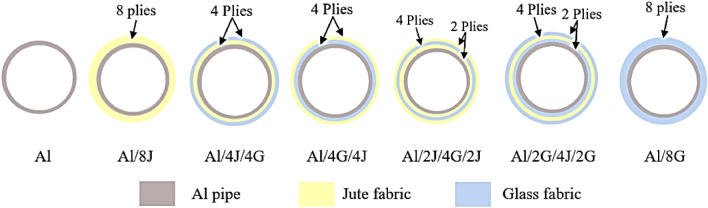
Stacking sequences of the fabricated hybrid pipes.

Wet warping by hand lay-up method was nominated to manufacture the test specimens, Fig. 2. Due to its ease and low requirements, this manufacturing method was employed by different investigators in many scholarships^4,5,27–30^. The manufacturing process steps can be summarized as follows:

1. Hand mixing and swirling of epoxy and its hardener took around 5 min. The mixture was uniformly added to the fabrics (Fig. 2a). The saturated fabrics were wrapped over a 50 mm treated aluminum pipe with (Fig. 2b).
2. The constructed pipes require “7” days at 25 $^\circ$C for full curing according to the matrix manufacturer recommendations^31^. The fabricated pipes were visually checked for imperfections after curing (Fig. 2c). The consolidated pipes were cut into 100 mm length (Fig. 2d).
3. Eight fabric plies with different orders were wrapped over the Al-pipes as shown in Fig. 3. Geometrical dimensions of test specimens are declared in Table 3.

**Table 3:** Geometrical descriptions of the fabricated metal-composite hybrid pipes.

Specimen code	Average specimen thickness (t), mm	Average specimen length (L), mm	Average specimen mass (M), g	Cost of each specimen, $	Normalized value of specimen cost^a^
Al	2.00	97.4	96.20	0.4810	1.0000
Al/8J	8.60	99.0	161.08	0.6218	1.2927
Al/4J/4G	4.33	98.5	141.55	0.5652	1.1751
Al/4G/4J	4.29	99.4	137.55	0.5572	1.1585
Al/2J/4G/2J	4.36	98.0	136.45	0.5550	1.1539
Al/2G/4J/2G	6.54	98.5	138.76	0.5596	1.1635
Al/8G	4.06	99.2	125.50	0.5396	1.1218

^a^Cost of the specimen divided by the cost of Al-specimen.

To ensure a strong bonding between Al-pipes, jute, and E-glass fabrics, Al-pipes underwent mechanical and chemical treatments. Firstly, Al-pipes were experienced to mechanical treatment by being rinsed with acetone, then smoothed down with # 400-grit sandpaper, then washed with distilled water, and finally dried in an oven. Secondly, mechanically treated Al-pipes were acid washed using HCl with 11% volumetric concentration for 30 min at room temperature. Then Al-pipes were submerged for 5 min at 70 °C in a 5 wt% NaOH solvent. Finally, treated Al-pipes were washed and dried to be used in manufacturing of hybrid composites^32^. This technique was adapted by many investigators^33–37^ who confirmed the success of this technique in enhancing the bond between metal and polymer interface.

### Testing protocol for determining crashworthiness indicators

**Figure 4:**
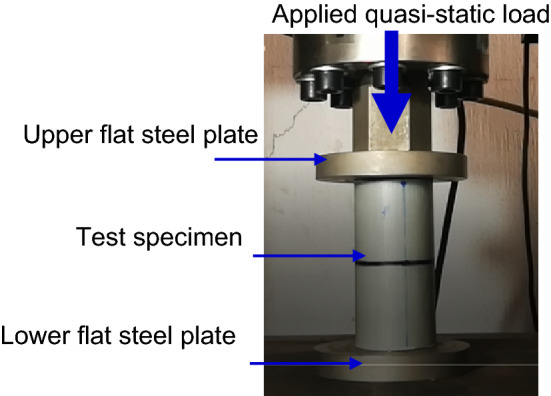
Experimental setup used in this work.

**Figure 5:**
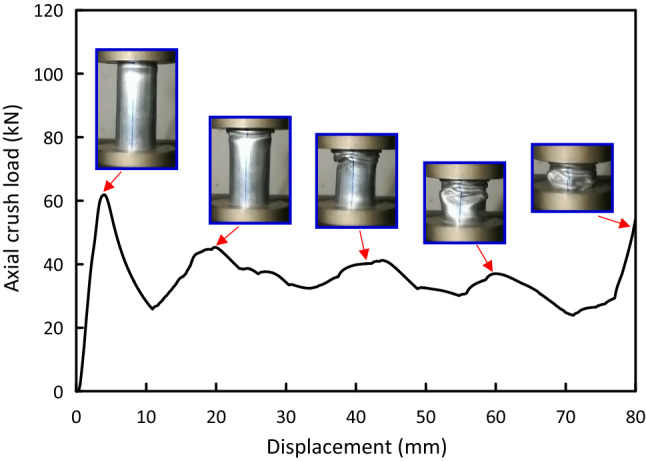
Load–displacement and crushing history for Al test specimen.

**Figure 6:**
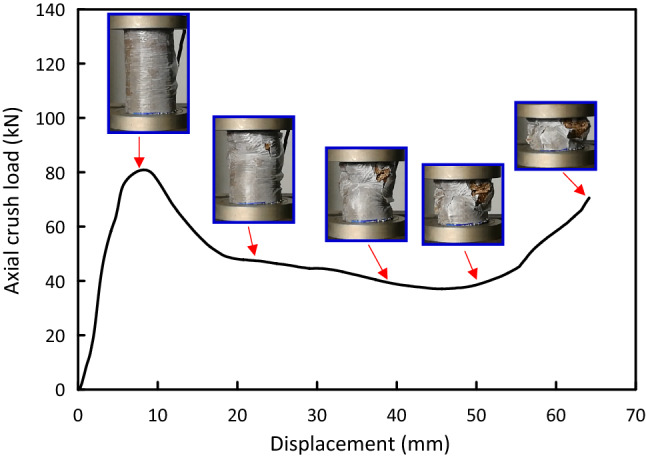
Load–displacement and crushing history for Al-8J test specimen.

**Figure 7:**
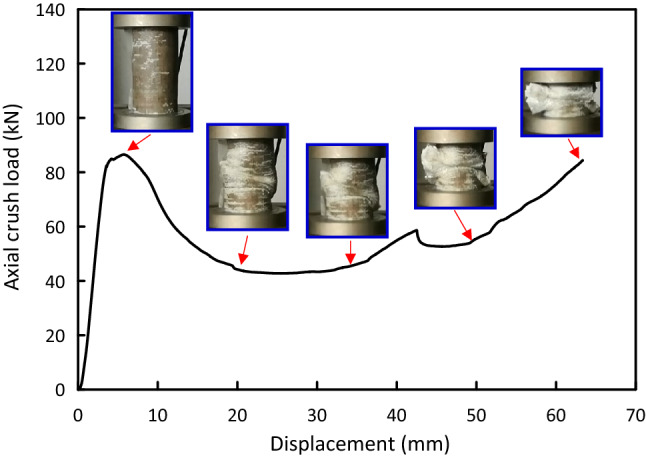
Load–displacement and crushing history for Al/4J/4G test specimen.

**Figure 8:**
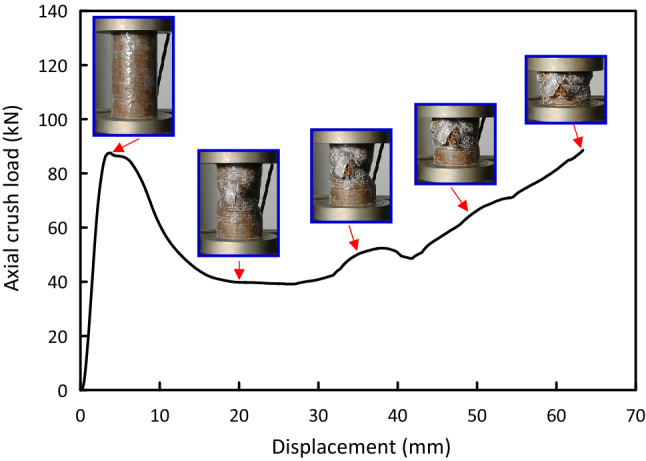
Load–displacement and crushing history for Al/4G/4J test specimen.

**Figure 9:**
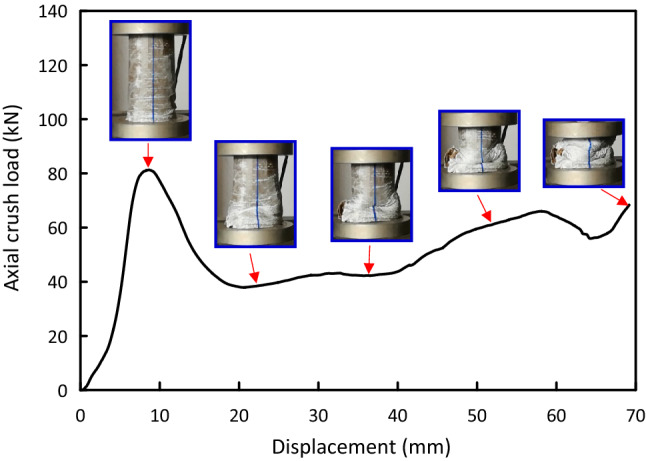
Load–displacement and crushing history for Al/2G/4J/2G test specimen.

**Figure 10:**
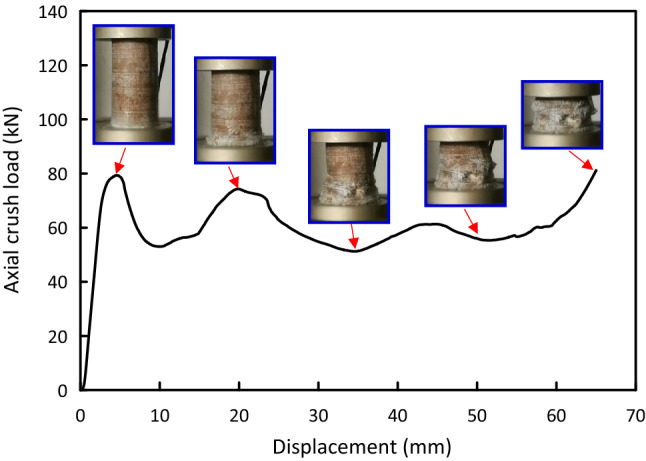
Load–displacement and crushing history for Al/2J/4G/2J test specimen.

**Figure 11:**
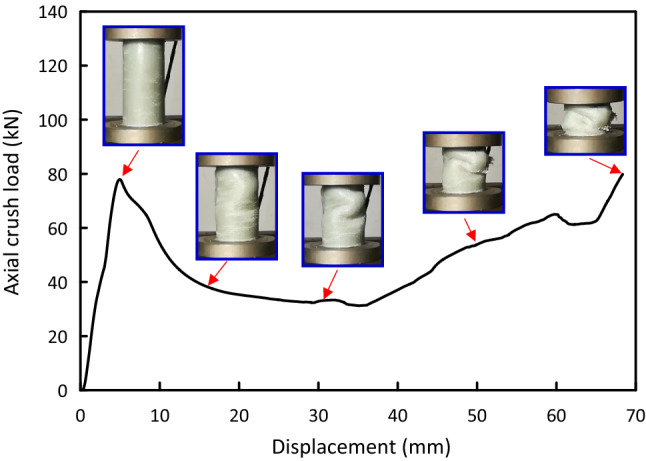
Load–displacement and crushing history of the Al/8G test specimen.

**Figure 12:**
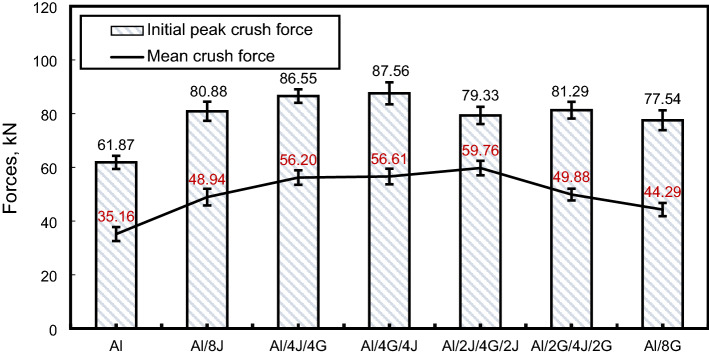
Initial and mean crushing loads for tested specimens.

**Figure 13:**
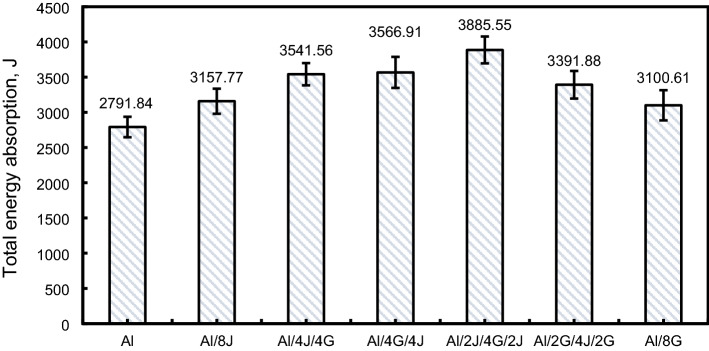
Total energy absorption for tested specimens.

**Figure 14:**
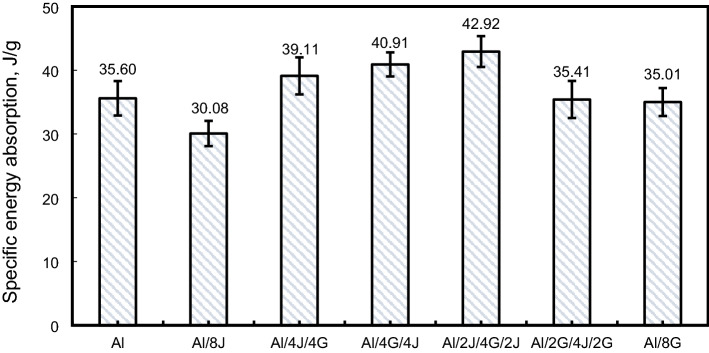
Specific energy absorption for tested specimens.

**Figure 15:**
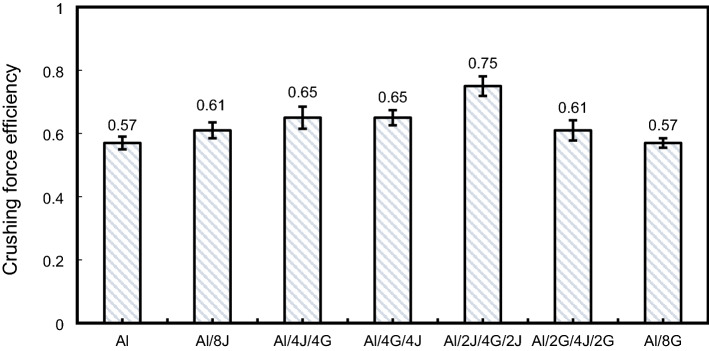
Crushing force efficiency for tested specimens.

100 kN universal testing machine (Type: Jinan WDW, China) was selected to perform the quasi-static tests at 10 mm/min crosshead speed. Figure 4 shows the experimental setup used in this work. Test specimens were positioned between two flat steel plates that were parallel to one another before the test began. Automatic data acquisition system was directly implemented to record load–displacement data. Many authors in their studies about crashworthiness confirmed this method^38–40^. The deformation histories of test specimens were tracked and reported. Load–displacement curves for three specimens in each case were recorded, and the average for the three curves was provided and drawn. The specimen whose curve is closer to the average curve was considered the most representative and was presented in Figs. 5, 6, 7, 8, 9, 10 and 11 as shown in the revised copy. Whilst the data shown in Figs. 12, 13, 14 and 15 represents the average values. The load–displacement curves produced can be used to quantify the performance of crashworthy metal-composite specimens. The following are the crushing critical parameters: peak crushing load (${\mathrm{F}}_{\mathrm{ip}}$), mean crushing load (${\mathrm{F}}_{\mathrm{m}}$), total energy absorption ($\mathrm{U})$, specific energy absorption $\left(\mathrm{SEA}\right),$ and crush force efficiency $\left(\mathrm{CFE}\right)$

- Peak crushing load (${\mathrm{F}}_{\mathrm{ip}})$ is recorded directly from the obtained load versus displacement plot. It's advised that it be compact enough to stop the energy absorber from imparting the force of the collision to the car^29^.
- Total energy absorption ($\mathrm{U})$ shows how much energy was wasted during collision process, Eq. (1).
  1 $$\mathrm{U}= {\int }_{0}^{{\updelta }_{\mathrm{max}}}\mathrm{ F}\left(\updelta \right)\mathrm{ d\delta },$$
  where, $\mathrm{F}\left(\updelta \right)\mathrm{ and }{\delta }_{\mathrm{max}}$ are the immediate crushing force and the entire crushing displacement, respectively.
- Mean crushing load (${\mathrm{F}}_{\mathrm{m}}$) can be determined by the total crush displacement and absorbed energy.
  2 $${\mathrm{F}}_{\mathrm{m}}= \frac{{\int }_{0}^{{\updelta }_{\mathrm{max}}}\mathrm{ F}\left(\updelta \right)\mathrm{ d\delta }}{{\delta }_{\mathrm{max}}}.$$
- Crush force efficiency $\left(\mathrm{CFE}\right)$ is the ratio between mean crushing load and peak crushing load. When the crush force efficiency percentage is high, the structure's effective EAC is also high.
  3 $$\mathrm{CFE}= \frac{{\mathrm{F}}_{\mathrm{m}}}{{\mathrm{F}}_{\mathrm{ip}}} \times 100.$$
- Specific energy absorption $\left(\mathrm{SEA}\right)$ is calculated by dividing the energy absorbed (U) by the energy absorber's mass (${\mathrm{m}}_{\mathrm{c}}$):
  4 $$\mathrm{SEA}=\frac{\mathrm{U}}{{\mathrm{ m}}_{\mathrm{c}}},$$
  5 $${\mathrm{m}}_{\mathrm{c}}=\left(\frac{\mathrm{M}}{\mathrm{L}}\right){\delta }_{\mathrm{max}},$$

where $\mathrm{M}/\mathrm{L}$ is the mass of the energy absorber per unit length.

## Results and discussion

### Load versus displacement plots and deformation history

Load versus displacement plots and the deformation histories for J/G reinforced epoxy over wrapped $(\mathrm{Al})$ pipes are shown in Figs. 5, 6, 7, 8, 9, 10 and 11. Results, declared in Figs. 5, 6, 7, 8, 9, 10 and 11, are for the most representative specimen for each configuration.

#### Al specimen

It is clear from Fig. 5 that $(\mathrm{Al})$ specimen behaves linear till it approaches the peak load of 61.87 kN at 3.97 mm then there is a sudden decrease to 25.23 kN at 10.32 mm. With increasing the displacement, pure $(\mathrm{Al})$ specimen regularly plastically deforms and yields high oscillations’ amplitude load-displacement profile in post-crash area till it reaches the start of the densification at about 79.41 mm. The crushing history for $(\mathrm{Al})$ pipe display folding and overall buckling of the pipe.

#### Al/8J specimen

It is clear from Fig. 6 that Al/8J specimen behaves linear till it approaches 80.88 kN at 8.06 mm, then there is a abrupt load declines to 49.65 kN at 18.56 mm. The load drop is accompanied with matrix cracking starting to happen at the pipe higher side due to stress concentration. The load–displacement plot then began to oscillate slightly in the post-crush stage until it reaches the start of the compaction area at 64.65 mm. The load quickly rises at the start of the compaction area. This outcome is in line with what was noted by Abdewi et al.^41^. Twisting and folding can be noticed. As a result of the fiber buckling, pipe overall buckling was recorded.

#### Al/4J/4G specimen

Load versus displacement plot and distortion history for Al/4J/4G specimen under quasi-static load are shown in Fig. 7. The pipe has a linear behavior till it approaches ${\mathrm{F}}_{\mathrm{ip}}$ of 86.55 kN at 5.74 mm, then a sharp drop in load to approximately 46.59 kN at 18.41 mm. After that, low fluctuations in the post crush zone were observed till the beginning of the densification zone at 65.01 mm. Wrinkling, wall bending, and global buckling accompanied with cracking can be noticed.

#### Al/4G/4J specimen

Figure 8 indicates the force versus movement response and crushing history for Al/4G/4J specimen attained from quasi-static compression test. The pipe has a linear trend till it approaches ${\mathrm{F}}_{\mathrm{ip}}$ of 87.57 kN at 3.76 mm, followed by a severe load drop at about 46.19 kN. After load drop, the load versus displacement plot oscillated in the post-crush zone until it enters the compaction zone's beginning at 63.01 mm. Matrix cracking at the bottom of the pipe can be noticed.

#### Al/2J/4G/2J specimen

Figure 9 illustrates the load versus displacement plot and the deformation history for Al/2J/4G/2J specimen attained from quasi-static test. The pipe has a line direction till it approaches ${\mathrm{F}}_{\mathrm{ip}}$ of 79.33 kN at 4.59 mm, the load–displacement curve oscillated in the post crush zone around the mean load until it enters the compaction zone's beginning at 65.01 mm. Matrix cracking at the bottom of the pipe can be noticed.

#### Al/2G/4J/2G specimen

Figure 10 demonstrates the load versus displacement plot and the deformation history for Al/2G/4J/2G specimen. The pipe performs linearly till it approaches ${\mathrm{F}}_{\mathrm{ip}}$ of 81.29 kN at 8.59 mm, followed by a sharp load drop at about 20.89 kN. After load drop, the load–displacement curve oscillated in the post crush stage till it reaches the beginning of the compaction zone at 70.29 mm. Matrix cracking at the bottom of the pipe can be noticed.

#### Al/8G specimen

Load versus displacement curve and distortion history for Al/8G specimen are displayed in Fig. 11. It was observed from that the pipe behaves linearly till it moves towards a load of 77.54 kN at 4.95 mm, followed by a sharp load drop to nearly 32.31 kN at 18.37 mm. After load drop, the load versus displacement plot oscillated in the post crush zone till the beginning of the densification zone at 72.00 mm. Global buckling with fiber fracture can be noticed for Al/8G specimen.

### Crashworthiness parameters

Table 4 indicates the crashworthiness parameters for all metal/polymer tested pipes. It also shows the repeatability of all data. It is clear that coefficient of variation (CV) of all results is less than 10% which confirms the repeatability of the results and reflect its visible accuracy.

**Table 4:** Crashworthiness parameters all metal/polymer tested pipes.

Specimen code	Sample No	${\mathrm{F}}_{\mathrm{ip}}$, kN	${\mathrm{F}}_{\mathrm{m}}$, kN	U, J	SEA, J/g	CFE
Al	1	64.84	37.84	2986.84	39.23	0.58
	2	58.87	31.65	2751.70	34.67	0.54
	3	61.90	35.99	2636.98	32.90	0.58
	Avg	61.87	35.16	2791.84	35.60	0.57
	SD	2.44	2.60	145.61	2.71	0.02
	CV, %	3.94	7.39	5.22	7.61	3.51
Al/8J	1	76.72	44.58	3145.98	29.43	0.58
	2	85.41	51.43	2945.75	28.04	0.60
	3	80.37	50.81	3381.58	32.77	0.63
	Avg	80.88	48.94	3157.77	30.08	0.60
	SD	3.56	3.10	178.12	1.98	0.02
	CV, %	4.40	6.33	5.64	6.58	3.33
Al/4J/4G	1	83.21	59.82	3700.02	42.74	0.72
	2	87.18	55.43	3597.33	38.93	0.64
	3	89.26	53.35	3327.33	35.66	0.60
	Avg	86.55	56.20	3541.56	39.11	0.65
	SD	2.51	2.70	157.21	2.90	0.04
	CV, %	2.90	4.27	4.44	7.41	6.15
Al/4G/4J	1	82.41	54.02	3337.96	38.33	0.66
	2	87.88	60.64	3498.73	41.66	0.63
	3	92.39	55.17	3864.04	42.74	0.66
	Avg	87.56	56.61	3566.91	40.91	0.65
	SD	4.10	2.90	220.11	1.88	0.01
	CV, %	4.68	5.12	6.17	4.60	1.54
Al/2J/4G/2J	1	82.51	57.37	3714.26	39.76	0.70
	2	80.55	63.55	4151.36	45.64	0.79
	3	74.93	58.36	3791.03	43.36	0.78
	Avg	79.33	59.76	3885.55	42.92	0.75
	SD	3.20	2.70	190.56	2.42	0.04
	CV, %	4.03	4.52	4.90	5.64	5.33
Al/2G/4J/2G	1	85.66	50.07	3335.55	35.21	0.58
	2	79.65	52.43	3654.40	39.01	0.66
	3	78.56	47.14	3185.69	32.01	0.60
	Avg	81.29	49.88	3391.88	35.41	0.61
	SD	3.12	2.20	195.45	2.90	0.03
	CV, %	3.84	4.41	5.76	8.19	4.92
Al/8G	1	75.76	47.60	3327.03	38.11	0.63
	2	82.68	43.53	3161.53	33.91	0.53
	3	74.28	41.74	2813.27	33.01	0.56
	Avg	77.54	44.29	3100.61	35.01	0.57
	SD	3.70	2.45	214.11	2.20	0.04
	CV, %	4.77	5.53	6.91	6.28	7.02

#### Peak $\left({{F}}_{{i}{p}}\right)$ and mean $\left({{F}}_{{m}}\right)$ crushing loads

As revealed in Fig. 12, the lowest ${(\mathrm{F}}_{\mathrm{ip}})$ was recorded for $(\mathrm{Al})$ pipe with a value of 61.87 kN. Hybridizing $(\mathrm{Al})$ pipe with eight layers of jute/epoxy, and glass/epoxy gives an enhancement of, respectively, 30.73 and 28.56% in ${\mathrm{F}}_{\mathrm{ip}}$ of $(\mathrm{Al})$ pipe. ${\mathrm{F}}_{\mathrm{ip}}$ of Al/4J/4G, Al/4G/4J, Al/2J/4G/2J and Al/2G/4J/2G pipes are, respectively, about 1.40, 1.42, 1.28 and 1.31 times that of Al pipe. This means that hybridizing $(\mathrm{Al})$ with jute and glass has a visible positive effect on the value of ${\mathrm{F}}_{\mathrm{ip}}$.

Hybridizing $(\mathrm{Al})$ pipe with eight layers of jute/epoxy, and glass/epoxy gives an enhancement of, respectively, 38.91 and 39.68% in $({\mathrm{F}}_{\mathrm{m }})$ of $\left(\mathrm{Al}\right)$ pipe. ${\mathrm{F}}_{\mathrm{ip}}$ of Al/4J/4G, Al/4G/4J, Al/2J/4G/2J and Al/2G/4J/2G pipes are, respectively, about 1.60, 1.61, 1.70 and 1.42 times that of $(\mathrm{Al})$ pipe. This means that hybridizing $(\mathrm{Al})$ with J and G fibers has a visible positive effect on the value of ${\mathrm{F}}_{\mathrm{m}}$.

#### Total absorbed energy $\left({U}\right)$

As represented in Fig. 13, the highest energy absorption $\left(\mathrm{U}\right)$ was noted for Al/2J/4G/2J pipe with a value of about 3885.55 kJ, whereas the lowest $\left(\mathrm{U}\right) \mathrm{was}$ noticed for $(\mathrm{Al})$ pipe with a value of about 2791.84 kJ, with enhancement of 39.18% in $\left(\mathrm{U}\right)$ compared with pure $(\mathrm{Al})$ pipe. Also, $\left(\mathrm{U}\right)$ value of Al/8J, Al/4J/4G, Al/4G/4J, Al/2G/4J/2G and Al/8G pipes are, respectively, about 1.13, 1.27, 1.28, 1.21 and 1.11 times that of $\left(\mathrm{Al}\right)$ pipe. This means that hybridizing $(\mathrm{Al})$ with jute and glass has a visible positive effect on the value of $\left(\mathrm{U}\right)$.

#### Specific absorbed energy $\left({S}{E}{A}\right)$

As demonstrated in Fig. 14, the highest specific energy absorption $\left(\mathrm{SEA}\right)$ value was recorded for Al/2J/4G/2J pipe with a value of about 42.92 kJ/g, with enhancement of 20.56% in $\left(\mathrm{SEA}\right)$ compared with pure Al pipe. Whereas the lowest $\left(\mathrm{SEA}\right)$ was detected for Al-8J pipe with a value of about 30.08 kJ/g. $\left(\mathrm{SEA}\right)$ of Al/4J/4G, Al/4G/4J, Al/2G/4J/2G and Al/8G pipes are, respectively, about 1.10, 1.15, 0.99 and 0.98 times that of $\left(\mathrm{Al}\right)$ pipe. It is clear that wrapping hybrid jute/glass reinforced epoxy layers over $\left(\mathrm{Al}\right)$ pipe improves $\left(\mathrm{SEA}\right)$ of $(\mathrm{Al})$ pipes.

#### Crush force efficiency $\left({C}{F}{E}\right)$

As represented in Fig. 15. The highest $\mathrm{CFE}$ value was recorded for Al/2J/4G/2J pipe with a value of about 0.75, whereas the lowest $\mathrm{CFE}$ was detected for $(\mathrm{Al})$ and Al/8G pipe with a value of about 0.57. Hybridizing $(\mathrm{Al})$ pipe with eight layers of jute/epoxy 7.01 and % in $\mathrm{CFE}$ of $\left(\mathrm{Al}\right)$ pipe. $\mathrm{CFE}$ of Al/4J/4G, Al/4G/4J and Al/2G/4J/2G pipes are, respectively, about 1.14, 1.14, and 1.07 times that of $(\mathrm{Al})$ pipe. This means that hybridizing $(\mathrm{Al})$ with jute and glass has a visible positive effect on the value of $\mathrm{CFE}$.

### Failure mechanism

**Figure 16:**
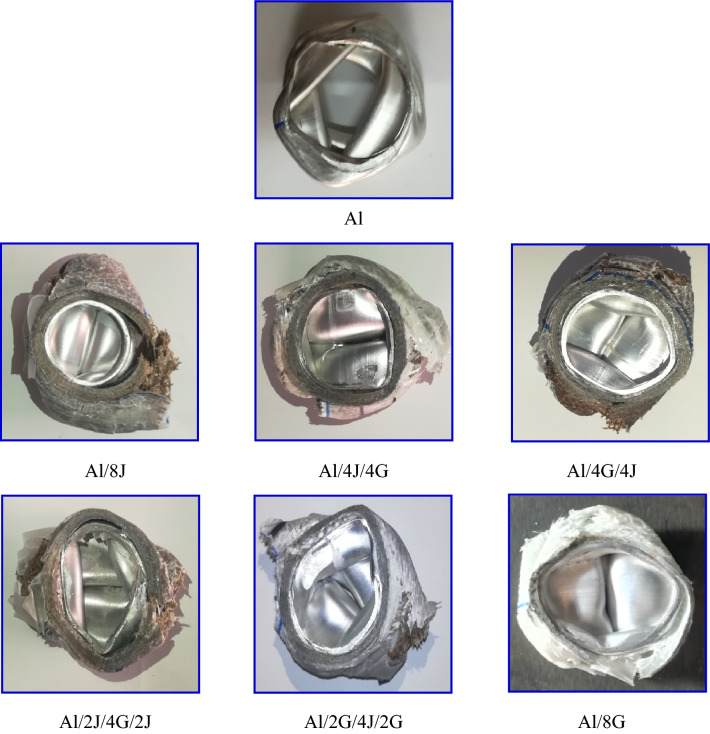
Top views for failed specimens.

Typically, energy absorbers are made to take in the crush energy. A crucial factor to consider when examining the EAC of metal-composite hybrid pipes is the failure mechanism^42^. Photos of typical samples of the crushed specimen's top views are included in Fig. 16. It is possible to see two damage modes. They fall into the following categories:

Approach I: Pristine $(\mathrm{Al})$ specimen recorded an axisymmetric or ring mode.

**Figure 17:**
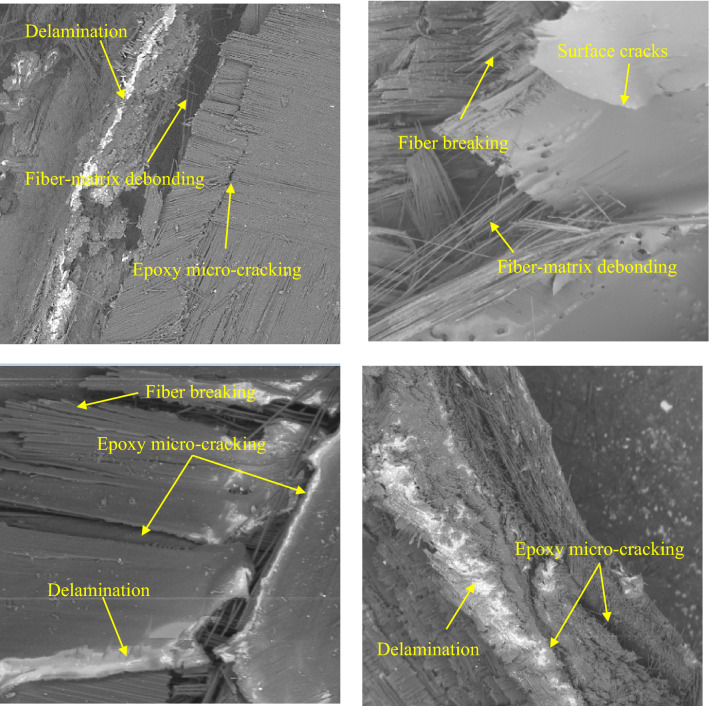
SEM for failed specimens.

Approach II: Initially, matrix macro-cracks formed and hybrid pipes began to buckle. The cracks then spread in a direction away from the pipe. Additional propagation of matrix cracking results in lamina bending, inner and outer folds’ formation, interlaminar delamination, fiber breaking and epoxy micro-cracking as shown in SEM images for failed specimens presented in Fig. 17.

### Cost analysis

**Figure 18:**
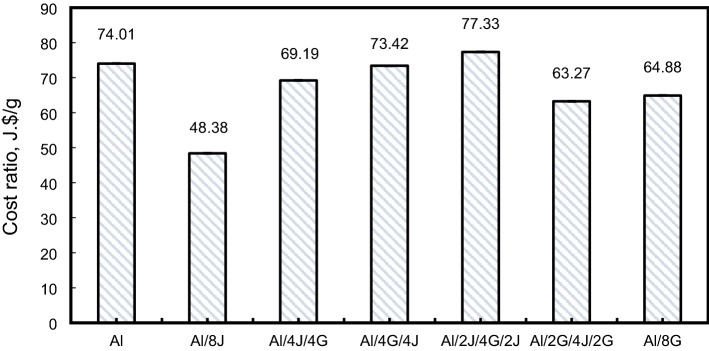
Cost ratio of the studied hybrid specimens.

When designing hybrid metal-composite pipes, cost is a critical factor influenced by both design and production factors. In this study, the prices of the used materials are 5.0 $/kg for Al 6063, 2.0 $/kg for E-glass woven fabric, 1.0 $/kg for jute woven fabric and 2.0 $/kg for the used epoxy resin. In this study, the cost ratio was evaluated as $$\left(\mathrm{SEA}\right)$$ divided by the cost of the pipe (Al, fiber, and matrix). It is obvious from Fig. 18 that Al/2J/4G/2J, Al, and Al/4G/4J pipes achieved the highest cost ratio with values of 77.33, 74.01, and 73.42J.$/g, respectively. Al/2J/4G/2J, Al, and Al/4G/4J pipes are the most effective ones and could be used in automotive applications as energy absorbing components. Table 3 includes the cost of each fabricated pipe and the normalized values of specimens’ cost.

### Comparison of SEA with published work

**Figure 19:**
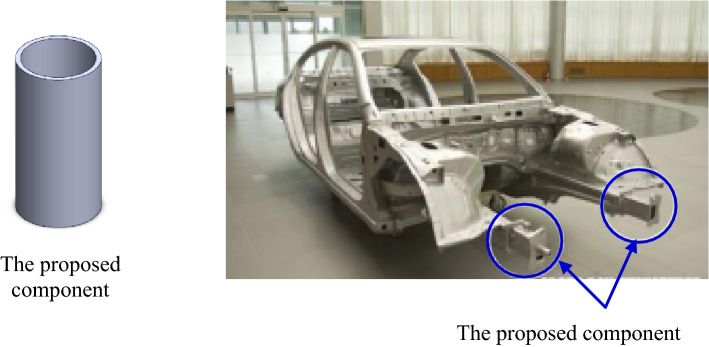
Recommended application for the proposed metal/polymer component and how to be incorporated.

Table 5 lists some earlier published data for $\left(\mathrm{SEA}\right)$ of energy absorbers made from natural/synthetic reinforced composites and metallic materials for evaluating the crashworthiness of the proposed material. It is clear from Table 4 that combining wrapping hybrid natural/synthetic fibers over $(\mathrm{Al})$ pipes can improves the crashworthiness performance of $(\mathrm{Al})$ energy absorbers. In addition, as compared with traditional metals, fiber reinforced composites, and hybrid pipes, the proposed pipes demonstrated improved crashworthiness performance, and as a result, the innovative energy absorber can be employed as energy absorbing components in the forward-facing of vehicle structures i.e., impact-resistant rods or a crash box and can also be adapted in airplanes fuselage. Crash boxes made from the proposed metal/polymer hybrid composites can be designed for a specific type of load for high-performance applications and safety equipment in transportation industries such as marine, aerospace, automotive industries as shown in Fig. 19.

**Table 5:** SEA for some metallic and fiber reinforcement composite tubes (labeled in the literature).

Material of the energy absorber tube	SEA (J/g)	References
Aluminum tube	32.41	Zhu et al.^43^
Carbon/epoxy	37.82	Zhu et al.^44^
Aluminum/carbon hybrid tube	22.81	–
Aluminum tube internally filled with CFRP tube	24.57	Zheng et al.^45^
CFRP tube internally filled with aluminum tube	23.80	–
Aluminum tube internally filled with CFRP tube filled with aluminum foam	25.77	–
CFRP tube internally filled with aluminum tube filled with aluminum foam	21.18	–
Hybrid kenaf/glass	5.64–14.39	Supian et al.^40^
(Jute/Kevlar)/epoxy	15.36–26.51	Albahash and Ansari^46^
Flax/epoxy	9.68–37.16	Yan and Chouw^47^
Wood	18–31.6	Guélou et al.^48^

## Conclusions

This article explores the effect of the layering sequences on the crashworthiness and damage mechanism of metal/polymer composite hybrid pipes. Circular pipes were prepared via hand wet wrapping procedure and subjected to axial loads. The following observations have been documented:

Hybridization and layering sequences process have a substantial effect on the crashworthiness and damage mechanisms of metal/polymer composite structures. The hybridization of $$(\mathrm{Al})$$ pipes with jute and glass reinforced epoxy layers leads to an increase in ($${\mathrm{F}}_{\mathrm{ip}}$$), ($${\mathrm{F}}_{\mathrm{m}})$$,$$(\mathrm{U})$$, and $$(\mathrm{CFE})$$. The highest ($${\mathrm{F}}_{\mathrm{ip}}$$) was recorded for Al/4G/4J with a value of 87.56. The highest ($${\mathrm{F}}_{\mathrm{m}})$$, $$(\mathrm{U})$$, $$\left(\mathrm{SEA}\right)$$, CFE, and cost ratio were recorded for Al/2J/4G/2J with, respectively, values of 59.76 kN, 3885.55 J and 42.92 J/g, 0.75, and 77.33 J.$/g. The exceptional capacity for absorbing energy, low weight and high cost ratio makes Al/2J/4G/2J suitable for use as energy dissipation components in automobiles.

Hybridizing $(\mathrm{Al})$ pipes with glass-jute reinforced epoxy changes the failure mechanism from axisymmetric or ring mode to buckling, matrix macro-cracks’ formation, crack propagation in the pipe's peripheral direction. Additional crack propagation leads to lamina bending and internal and external folds’ formation, delamination, fiber breaking, and fiber pull-out.

## Data Availability

The datasets generated during and/or analyzed during the current study are available from the corresponding author on reasonable request.
